# The Antarctic Circumpolar Current as a diversification trigger for deep-sea octocorals

**DOI:** 10.1186/s12862-015-0574-z

**Published:** 2016-01-04

**Authors:** Luisa F. Dueñas, Dianne M. Tracey, Andrew J. Crawford, Thomas Wilke, Phil Alderslade, Juan A. Sánchez

**Affiliations:** Department of Biological Sciences, Universidad de los Andes, A.A. 4976 Bogotá, Colombia; Department of Animal Ecology & Systematics, Justus Liebig University, Giessen, Germany; National Institute of Water and Atmospheric Research-NIWA, Wellington, New Zealand; Smithsonian Tropical Research Institute, Apartado, 0843-03092 Panama City, Republic of Panama; CSIRO Marine and Atmospheric Research, PO Box 1538, Hobart, Tasmania 7001 Australia

**Keywords:** Antarctic Circumpolar Current, Gene flow, Primnoid octocorals, Southern Ocean, Deep-sea, Statistical phylogeography

## Abstract

**Background:**

Antarctica is surrounded by the Antarctic Circumpolar Current (ACC), the largest and strongest current in the world. Despite its potential importance for shaping biogeographical patterns, the distribution and connectivity of deep-sea populations across the ACC remain poorly understood. In this study we conducted the first assessment of phylogeographical patterns in deep-sea octocorals in the South Pacific and Southern Ocean, specifically a group of closely related bottlebrush octocorals (Primnoidae: *Tokoprymno* and *Thourella*), as a test case to study the effect of the ACC on the population structure of brooding species. We assessed the degree to which the ACC constitutes a barrier to gene flow between northern and southern populations and whether the onset of diversification of these corals coincides with the origin of the ACC (Oligocene-Miocene boundary).

**Results:**

Based on DNA sequences of two nuclear genes from 80 individuals and a combination of phylogeographic model-testing approaches we found a phylogenetic break corresponding to the spatial occurrence of the ACC. We also found significant genetic structure among our four regional populations. However, we uncovered shared haplotypes among certain population pairs, suggesting long-distance, asymmetrical migration. Our divergence time analyses indicated that the separation of amphi-ACC populations took place during the Middle Miocene around 12.6 million years ago, i.e., after the formation of the ACC.

**Conclusion:**

We suggest that the ACC constitutes a semi-permeable barrier to these deep-sea octocorals capable of separating and structuring populations, while allowing short periods of gene flow. The fluctuations in latitudinal positioning of the ACC during the Miocene likely contributed to the diversification of these octocorals. Additionally, we provide evidence that the populations from each of our four sampling regions could actually constitute different species.

**Electronic supplementary material:**

The online version of this article (doi:10.1186/s12862-015-0574-z) contains supplementary material, which is available to authorized users.

## Background

Despite our growing understanding of the role of Antarctica in driving global climate regimes and regional patterns of marine diversity, the distribution and connectivity among deep-sea benthic populations across the Southern Ocean remain poorly known. This vast and remote region of the globe hosts a surprising diversity of organisms [1] that thrive under extreme environmental conditions. While many of the Southern Ocean species from shallower continental shelves were discovered and described in the early 20th Century [2], exploration vessels with state-of-the-art underwater equipment are now sampling deep-sea regions. Molecular methods have also aided tremendously in the discovery of new species and the inference of their evolutionary origins and population genetic structure [3, 4]. Despite this increased exploration, only an estimated one-quarter of the continental shelf fauna has been described [5, 6], and most deep-sea regions have never been explored. Consequently, more research is needed on these deep-sea frontiers in order to assess faunal diversity and endemism, and to understand the origin, distribution and maintenance of deep-sea populations in the Southern Ocean.

The Antarctic continent and its attending Southern Ocean are surrounded by the Antarctic Circumpolar Current (ACC), the largest and strongest current in the world [7]. The onset of the ACC is thought to have occurred at the Oligocene-Miocene boundary around 25 million years ago (Ma), when South America separated from Antarctica creating the Drake Passage [8]. The ACC is characterized by an extreme transition of temperature and by profound depths of over 1000 m; in some places even extending to the seabed at a depth of 4000 m [9]. The particular oceanographic conditions of the ACC likely promoted the biogeographic isolation of the Southern Ocean [10] by creating a natural marine barrier to genetic exchange [7]. Some marine benthic organisms are distributed across the ACC, however, suggesting some level of permeability and potential gene flow [11–13]. Therefore, the ACC could represent a distinctive biogeographical discontinuity, where few marine benthic organisms occur both north and south of this current [14]. Nevertheless, at present it remains unclear what factors determine whether the ACC acts as a strong barrier against gene flow for some organisms and yet remains permeable to others.

Gene flow is governed by the interaction of extrinsic environmental factors with intrinsic factors determined by the natural history of each species [15]. In the case of marine organisms, extrinsic factors such as light, temperature, pH and of course currents may promote or impede larval dispersal. Using oceanographic data, Clarke et al. [14] identified turbulent flow structures, called eddies, over a wide range of scales in the ACC. These eddies can have warm-core or cold-core rings, transporting water and larvae from sub-Antarctic to Antarctic waters, and vice versa. Anthropogenic mechanisms could also carry organisms across the ACC, e.g., increasingly frequent ship transport that may carry encrusting organisms on their hulls or free-swimming organisms in ballast waters [16]. Factors intrinsic to the life history of marine organisms may also determine levels of gene flow, especially those traits related to dispersal ability [10], e.g., brooder versus broadcast spawners. The latter go through a pelagic larval stage that can span from a few days to several months [17], facilitating possible long-distance dispersal. In contrast, brooders incubate their larvae that then settle and metamorphose at short distances from their parents [17, 18]. Given such variation in reproductive strategies and developmental mode among benthic invertebrates, we expect that with a decreasing length of the pelagic larval phase (or for brooder species without pelagic larvae), there will be an increase in genetic differentiation [19, 20].

**Fig. 1:**
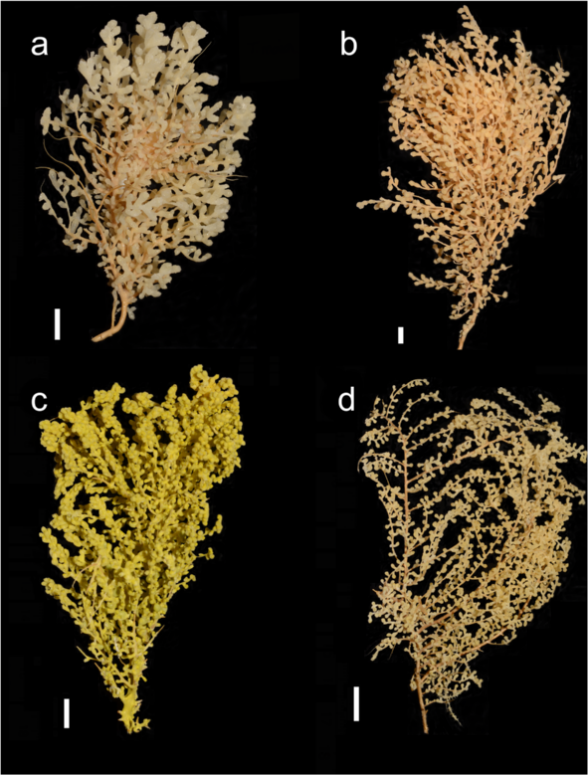
Photographs of the bottlebrush deep-sea octocoral colonies from the South Pacific and Southern Ocean. Each letter represents an example of the colonies from each population: **a**- New Zealand, **b**- Tasmania, **c**- Macquarie Ridge, **d**- Antarctica. The bar next to each colony corresponds to a 1 cm scale

**Fig. 2:**
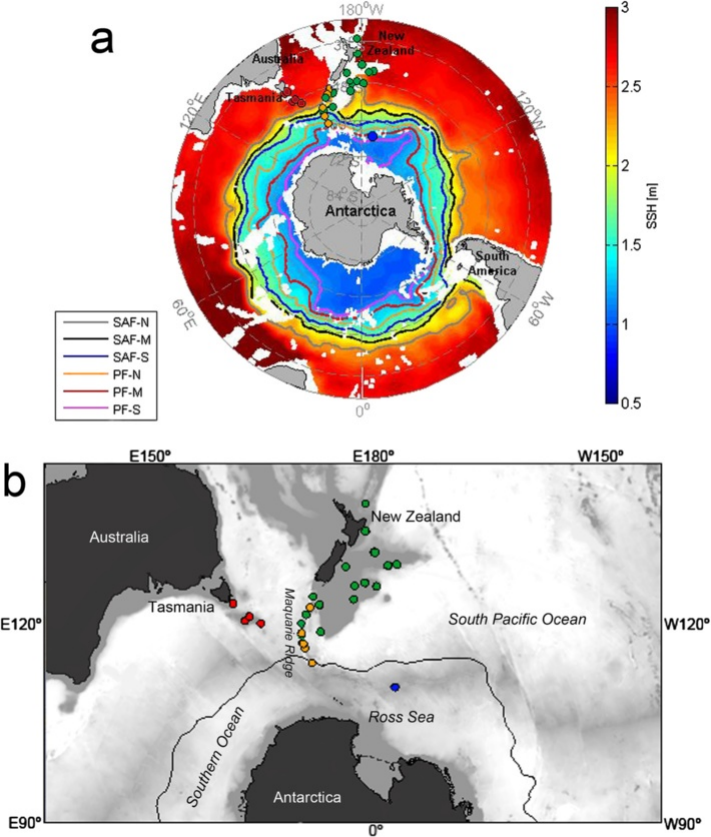
Geographic distribution of samples of bottlebrush deep-sea octocorals from the South Pacific and Southern Ocean. Samples from Tasmania are shown in red, samples from New Zealand in green, samples from the Macquarie Ridge in orange, and samples from Antarctica in blue. **a**- ACC front positions mapped using Sea Surface Height (SSH) overlaying Southern Ocean mean SSH. Fronts showed are sub-Antarctic Front (SAF-N, SAF-M, SAF-S); Polar Front (PF-N, PF-M, PF-S); -N, northern branch; -M, middle branch; -S, southern branch. Fronts are identified by their SSH signal following the methods described in Sokolov and Rintoul [108]. Blank spaces indicate bathymetry shallower than 2500 m. **b**- Zoom on the localities from this study. In this case bathymetry is shown in grey tones, where darker ones represent shallower waters. The black line surrounding Antarctica shows the position of the Polar Front

One brooding deep-water taxon occurring across the ACC (Fig. 1) is the bottlebrush-like octocoral, *Tokoprymno maia* Bayer [21] (Octocorallia: Primnoidae). *Tokoprymno maia* was originally described from sub-Antarctic waters (southeastern Pacific Basin, 54°49′S 129°48′W) at a depth of 549 m [21] but is widely distributed in the southern Pacific Ocean. Recent deep-sea voyages have also located this species on seamounts around New Zealand and south of Tasmania (Australia) at depths of 350 m to 1700 m (Fig. 2). Preliminary morphological and genetic analyses, part of which will be shown in this study, indicate that this primnoid octocoral could comprise a complex of species that includes nominal *T. maia* as well as populations from another taxonomic unit from sub-Antarctica. The later closely resembles a species of the sister genus *Thouarella* Gray, 1870 [22]; namely *T. viridis* Zapata-Guardiola and López-González, (2010) (Dueñas et al., unpublished observations). However, there are a few differences between *T. viridis* and our *Thouarella*-like samples, namely: sculpturing on the surface of the coenenchmal sclerites; the colour of the colonies; and the distribution, as *T. viridis* has only been reported from the sub-Antarctic waters in the South Atlantic. Nevertheless, despite the morphological differences between *T. maia* and the *Thouarella*-like colonies, our molecular analyses clearly show that they share haplotypes. Therefore, we consider them to be closely related to the *T. maia* samples used in this study and they will be treated as part of a complex that will be referred to in the text as ‘the primnoid bottlebrush octocorals’. Data presented below support this simplifying hypothesis. A manuscript formally addressing this taxonomic puzzle will follow.

In this study we use these particular primnoid bottlebrush corals as a test case to assess the effect of the world’s largest and strongest ocean current on the population structure of brooding amphi-ACC species. Our specific questions are:

1. Does the ACC constitute a barrier to gene flow between northern and southern populations? Our null hypothesis is that the ACC does not currently act as a barrier, resulting in a panmictic population with no genetic structure between localities. This prediction will be tested through phylogeographical analyses of multiple *a priori* migration models.
2. If the former hypothesis is rejected, does the origin of the ACC during the Oligocene-Miocene boundary coincide with the onset of diversification within the primnoid bottlebrush corals? Our null hypothesis is that the ACC constitute an old barrier, in which case the onset of divergence between populations north and south of the current should corresponded to the approximate date of the onset of the ACC around 25 Ma. This prediction will be tested using a molecular clock approach.

Assessing the mechanisms underlying the population genetic structure of deep-sea species could help shed light on the role of marine barriers as diversification triggers and how connectivity is maintained in marine populations, particularly across large biogeographic features such as the ACC.

## Results

### Phylogenetic analyses

DNA samples from 80 specimens were successfully amplified and sequenced for two rDNA nuclear regions: the internal transcribed spacer 2 (ITS2) and 28S. The data matrix had a total of 927 characters from which 497 were parsimony-informative.

**Fig. 3:**
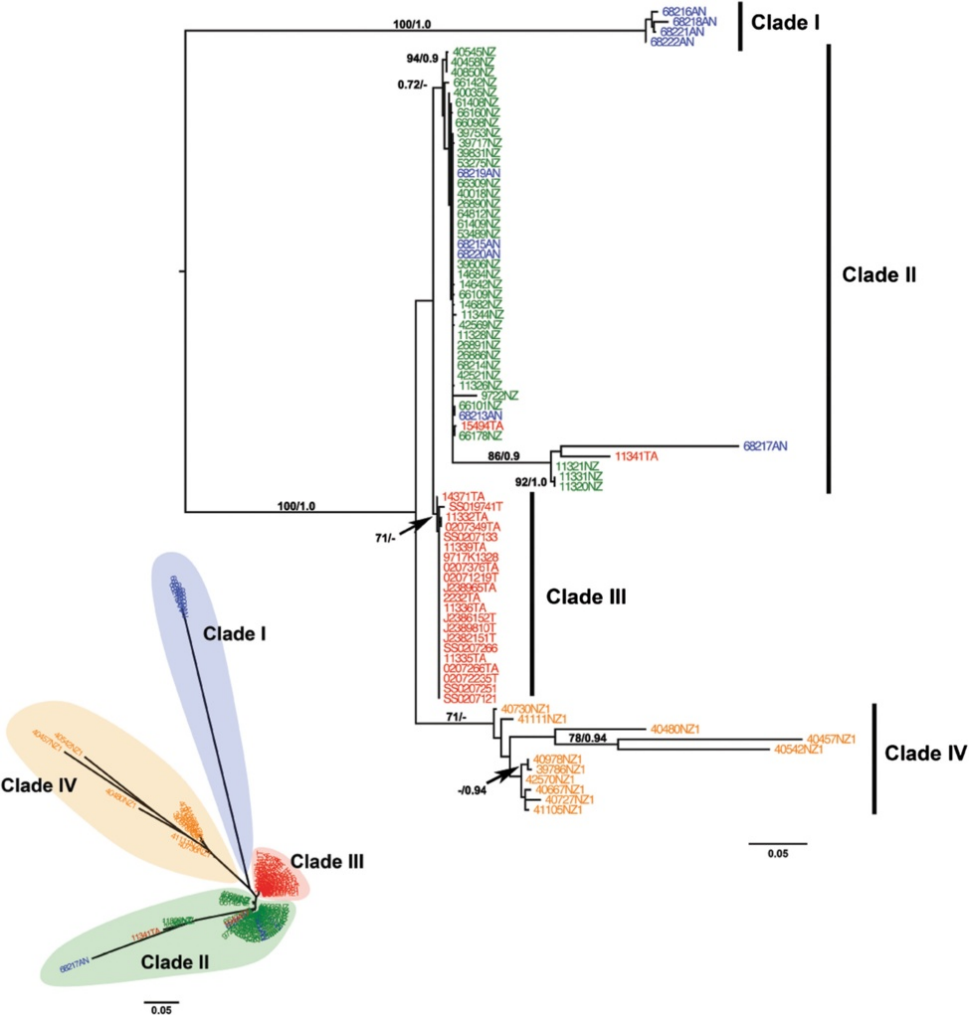
Maximum Likelihood tree based on concatenated ITS2 and 28S gene sequences. The lower left figure shows the un-rooted tree while the figure on the right shows the mid-point rooted topology for the primnoid bottlebrush octocorals from the South Pacific and Southern Ocean. Support values are provided if higher than 70 % for ML and 0.9 for BI (actual BI trees are not shown). The colours of the sample names correspond to the localities: red for Tasmania, green for New Zealand, orange for Macquarie Ridge, and blue for Antarctica

Topologies inferred using Maximum Likelihood (ML) and Bayesian Inference (BI) were nearly identical, with some variation in support values. Figure 3 displays the resulting unrooted and mid-point rooted topologies. The phylogeny showed a basal split of lineages across the ACC, corresponding to southern samples (Clade I) and northern samples (Clades II-IV). We inferred four well-supported clades, where Clade I contained only samples from Antarctica (Fig. 3). Clade II contained mainly samples from New Zealand, plus five from Antarctica and two from Tasmania. Clade III contained only samples from Tasmania. Finally, Clade IV was comprised exclusively of samples from the Macquarie Ridge, a long seamount chain south of New Zealand (Fig. 2).

Both rDNA gene regions showed substantial among-individual variation when applied to the study of deep-sea bottlebrush octocorals, but no evidence of intra-individual variation. 28S showed genetic divergences within clades ranging from 0.03 to 6.79 % (uncorrected p-distances), and between clades from 3.97 to 18.47 %. ITS2 showed higher pairwise distances in comparison to 28S, i.e., within-clade distances ranging from 0.17 to 3.00 % and between-clade distances from 4.98 to 33.55 %. Total genetic divergences based on concatenating the two nuclear loci ranged from 0.1 to 6.1 % within clades and from 1.9 to 19.1 % between clades.

### Phylogeographical analyses

Using the Bayes Factor approach we found that the four-population model had the highest marginal likelihood (Additional file 1) as estimated by thermodynamic integration, thus supporting the phylogenetic premise of separating the Macquarie Ridge population from the New Zealand population. Therefore, we recognized Tasmania, Antarctica, New Zealand and the Macquarie Ridge as four distinct populations for all posterior analyses.

**Fig. 4:**
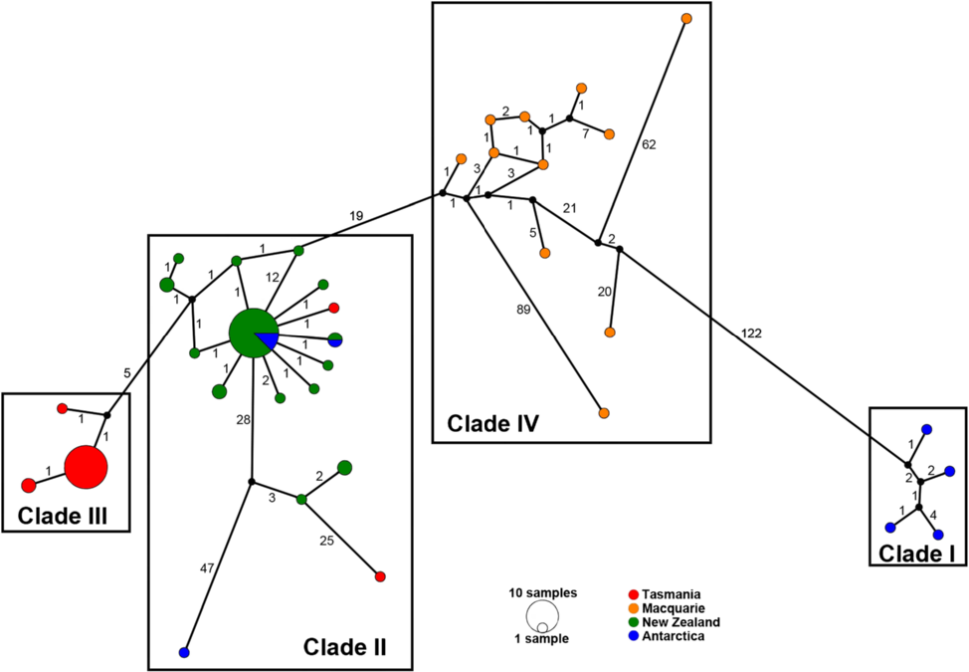
Median-joining network based on 80 concatenated ITS2 and 28S sequences from the bottlebrush deep-sea octocorals. A coloured circle represents each unique haplotype and its area is proportional to its relative frequency in the data set. Colours indicate the proportion of individuals sampled in each region. Small black circles represent intermediate haplotypes, and numbers along branches represent the number of substitutions among haplotypes. The structure of the clades corresponds to the one in Fig. 2

The haplotype relationships show the same clades as in the inferred phylogenetic tree (Fig. 4). There are four main clades, where Clade I has four haplotypes all from Antarctica. Clade II, composed mainly of samples from New Zealand, has a main dominant haplotype shared with Antarctica and other closely related haplotypes from Antarctica and Tasmania. Clade III is represented by one dominant haplotype, composed exclusively by samples from Tasmania. Finally, Clade IV is represented exclusively by individuals from the Macquarie Ridge where there are no dominant haplotypes (Fig. 4).

Preliminary population genetic analyses showed that Hd and *θ*_W_ were higher in Antarctica and the Macquarie Ridge (Table 1). Nucleotide diversity was highest in Antarctica, with New Zealand and Tasmania showing the lowest value of 0.01 (Table 1). The AMOVA that grouped all localities as one population (i.e., no ACC barrier) showed that the percentage of variation among localities is slightly higher (56.50 %) than within localities, with significant structure (*F*_ST_ = 0.57, *P* < 0.001). On the other hand, the AMOVA that assumed that the sub-Antarctic localities (Tasmania, New Zealand, and Macquarie Ridge) were one population (i.e., ACC as a barrier) also indicated a significant population structure (*F*_ST_ = 0.61, *P* < 0.001) across the ACC, with substantial variation partitioned among localities within populations (47.17 %; Table 2). Pairwise *F*_ST_ analyses showed significant population structure between all pairs of localities, with the most marked population structure found between Tasmania and Macquarie (*F*_ST_ = 0.61, *P* < 0.001), and the smallest between Antarctica and Macquarie (*F*_ST_ = 0.31, *P* < 0.001; Table 3).

**Table 1:** Genetic polymorphisms within populations of the bottlebrush octocorals from the South Pacific and Southern Ocean

Population	n	h	Hd	π	θw
Tasmania	23	6	0.46	0.01	0.03
Macquarie Ridge	11	11	1.00	0.06	0.08
New Zealand	37	14	0.68	0.01	0.02
Antarctica	9	7	0.92	0.13	0.10

The variables in the table represent: number of samples (n), number of haplotypes (h), haplotype diversity (Hd), nucleotide diversity per site (π), Watterson’s estimate of the population mutation rate per site (θ_W_)

**Table 2:** Analysis of Molecular Variance (AMOVA) implemented in Arlequin 3.5.1.3 [91]

Source of variation	d.f.	Sum of squares	Variance components	Percentage of variation	*F*_*SC*_	*F*_*ST*_	*F*_*CT*_
Among localities	3	1345.80	24.01	56.50			
Within localities	76	1404.52	18.48	43.50			
Total	79	2750.31	42.49			0.57***	
Among populations	1	366.82	6.31	13.44			
Among localities within populations	2	978.97	22.13	47.17			
Within localities	76	1404.52	18.48	39.39			
Total	79	2750.31	46.92		0.55***	0.61***	0.13

Four localities (Tasmania, Macquarie Ridge, New Zealand and Antarctica) of the primnoid bottlebrush octocorals from the South Pacific and Southern Ocean were assessed. The top section evaluates a grouping in which all collecting localities are part of one unique population (i.e., ACC as no barrier), while the bottom section evaluates a scenario in which the sub-Antarctic localities are grouped as one population (Tasmania, Macquarie Ridge, and New Zealand) compared to Antarctica (i.e., ACC as a barrier)

*** indicates *P* < 0.001

**Table 3:** *F*_*ST*_ values for pairwise comparisons among populations of primnoid bottlebrush deep-sea octocorals

	Tasmania	Macquarie Ridge	New Zealand	Antarctica
Tasmania	-	-	-	-
Macquarie Ridge	0.61***	-	-	-
New Zealand	0.49***	0.59***	-	-
Antarctica	0.36***	0.31***	0.35*	-

Comparisons based on concatenated ITS2 and 28S gene sequences*.* Sample sizes are given in Table 1

*0.01 < *P* <0.05; ***P < 0.001

Among eight distinct spatial migration models, two were highly supported by our log Bayes Factor (LBF) analyses: asymmetric migration from north to south (Model IV) and a stepping-stone model (Model VI), (Table 4). The worst two models both assumed that the ACC was a barrier (Model VII and Model VIII), i.e., Antarctica was isolated from the sub-Antarctic populations (Table 4).

**Table 4:** MIGRATE-N [23] runs showing the log-probability of the data given the model (marginal likelihood) for the following migration models: I) full migration between the four localities; II) migration from West to East; III) migration from East to West; IV) migration from North to South; V) migration from South to North; VI) a stepping-stone model; VII) ACC as a permanent barrier between north and south populations, with full migration between northern populations; VIII) ACC as a permanent barrier and stepping-stone between northern populations

	Model I	Model II	Model III
			
No. Parameters	16	10	10
Specification	****************	*000**00***0****	****0***00**000*
Bezier lmL	−5236.6250	−5223.4028	−5221.5584
LBF	−20.70	−7.48	−5.63
Choice	6	4	3
Posterior Prob.	<0.001	<0.001	<0.01
	Model IV	Model V	Model VI
			
No. Parameters	10	10	10
Specification	*0*0***000*0****	**0*0*0*****000*	**00****0**00*0*
Bezier lmL	−5216.5184	−5223.9708	−5215.9249
LBF	−0.59	−8.05	0.00
Choice	2	5	1
Posterior Prob.	0.36	<0.001	0.64
	Model VII	Model VIII	
			
No. Parameters	10	8	
Specification	***0***0***0000*	**00***00**0000*	
Bezier lmL	−5930.9088	−6300.2584	
LBF	−714.98	−1084.33	
Choice	7	8	
Posterior Prob.	<0.0001	<0.0001	

The first row represents the number of parameters used for a particular model. The second row shows the symbol specification for the migration matrix given to MIGRATE-N, where and asterisk represents an estimated parameter and a zero a non-estimated parameter. The third row provides the log marginal likelihood for each model obtained by thermodynamic integration. The forth row reports the log Bayes Factor (LBF). The row ‘Choice’ orders the models based on the LBF, and finally, the sixth row shows the probability for each model. Populations are labelled as follows: T for Tasmania, MQ for Macquarie Ridge, NZ for New Zealand, and A for Antarctica

**Fig. 5:**
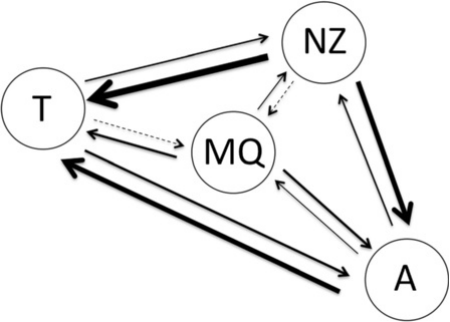
Graphical summary of relative unidirectional migration rates estimated from the best-supported model of population structure. The arrows show the estimates for the direction of migration between populations of the primnoid bottlebrush octocorals from the South Pacific and Southern Ocean, and the thicknesses are proportional to the mutation-scaled migration rate (M). Populations are labelled as follows: T for Tasmania, MQ for Macquarie Ridge, NZ for New Zealand, and A for Antarctica

MIGRATE-N reports mutation-scaled migration rates (*M = m/μ*), where *m* is immigration rate per generation and *μ* the mutation rate. The mutation-scaled migration rate represents the importance of within-population variation derived from migration events relative to mutation [23]. In the absence of reliable estimates of μ for ITS2 and 28S in octocorals, we graphically present estimates of the relative values of *M*, where the arrows show the direction of migration events and the thickness is proportional to the mutation-scaled migration rate (Fig. 5). Unidirectional migration rates were highest from New Zealand to Tasmania, from New Zealand to Antarctica, and from Antarctica to Tasmania. The lowest estimates were from Tasmania to the Macquarie Ridge, and from New Zealand to the Macquarie Ridge. Estimates and attending confidence intervals around *M* are given in Additional file 2.

### Divergence time estimation and ancestral character state reconstruction

**Fig. 6:**
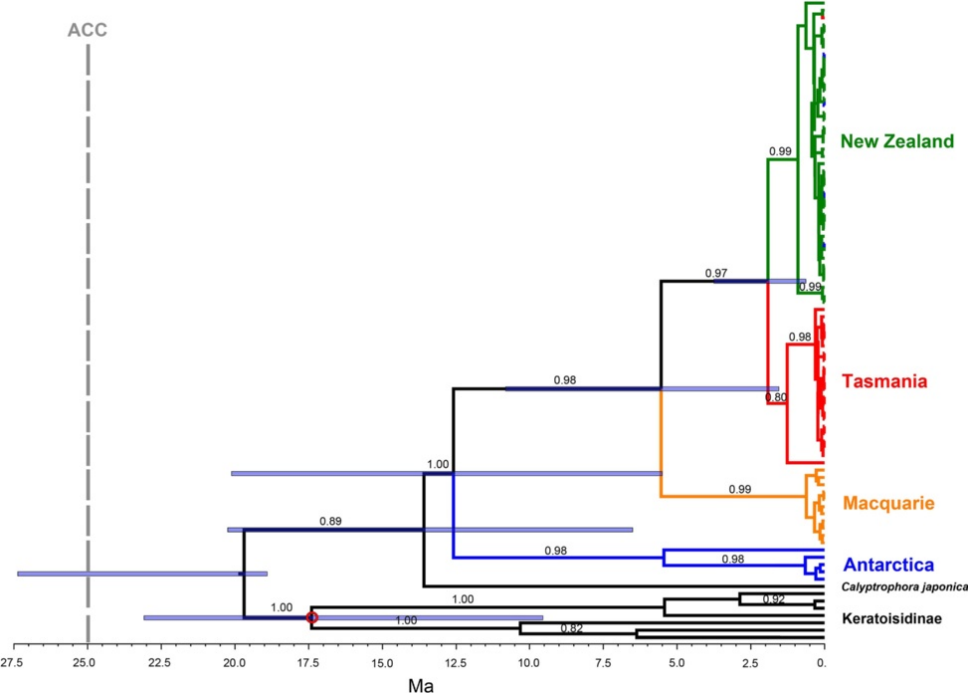
Timetree obtained using Bayesian MCMC phylogenetic analyses in BEAST 1.8 [82] based on ITS2 gene sequences. Values above branches are posterior probabilities above 0.8 for the main clades. The blue bars represent the 95 % highest posterior density (HPD) interval for the divergence time estimates, shown only for clades of interest. The red circle represents the calibrated node. See text for fossil calibration details. The vertical line represents the onset of the Antarctic Circumpolar Current (ACC), estimated at 25 Ma [8]

The stem age of bottlebrush octocorals was estimated at 13.7 Ma (95 % credible interval [C.I.] of 6.6–20.3 Ma) and the divergence between Antarctica and the northern populations (crown age) took place during the Middle Miocene 12.6 Ma (95 % C.I. 5.6–20.2 Ma; Fig. 6). Populations in the Macquarie Ridge separated from those in Tasmania and New Zealand around 5.5 Ma (95 % C.I. 1.6–10.9 Ma), and finally populations from Tasmania and New Zealand separated from each other around 1.8 Ma (95 % C.I. 0.7–3.9 Ma).

The ancestral area reconstruction was largely unresolved. Both the maximum likelihood reconstruction and the stochastic mapping showed probabilities of roughly 0.25 for each possible state at the ancestral node (Node A; Additional files 1, 3 and 4). Node B, the common ancestor of Macquarie Ridge plus New Zealand, showed a similarly ambiguous pattern under either reconstruction methods. Finally, for Node C, the common ancestor of Tasmania plus New Zealand, the highest probability was for a Tasmanian ancestor (Additional files 3, 4 and 5).

## Discussion

This study represents the first attempt to explore the roll of the ACC as a potential barrier to gene flow in deep-sea octocorals from the Southern Pacific Ocean and Southern Ocean. The phylogenetic analyses strongly suggest a phylogenetic break between amphi-ACC populations. Finding two distinct, reciprocally monophyletic clades in the vicinity of New Zealand was wholly unexpected. The diversification of the bottlebrush deep-sea octocorals was estimated at 6.6–20.3 Ma, well after the formation of the ACC at 25 Ma. Thus, the initial isolation of Antarctic populations was likely driven by other oceanographic changes during the Middle Miocene, or else the process of isolation by the ACC perhaps took some ten million years. Despite the high genetic structuring among our four regional populations, we uncovered shared haplotypes among certain population pairs, suggesting long-distance migration. Recent migration between populations north and south of the ACC was also seen, where population genetics analyses revealed a complex scenario of isolation with asymmetric gene flow. Consequently, the ACC has not served as an impermeable gene flow barrier for these brooder corals.

### The ACC as a barrier to gene flow

The phylogenetic analyses revealed deeply structured geographic clades, combined with some clear cases of haplotype sharing, particularly between New Zealand and Tasmania, as well as between New Zealand and Antarctica. These migrant individuals were independently extracted, PCR amplified and sequenced multiple times in separate laboratories to rule out contamination as an explanation. Thus, our data clearly demonstrate migration events between these localities, probably more recent than 100 thousand years ago (Ka). Additional analyses using multilocus coalescent approaches would be useful to infer the exact age of such recent events, and, although this is not the scope of this study, we do know that since 30 Ka the Southern Ocean regime has fluctuated dramatically. Any of these oceanographic fluctuations may have promoted dispersal by brooding octocorals among our four populations. Bostock et al. [24] used microfossil assemblages to assess the change in oceanographic features and showed shifts in sea surface temperatures (SST), position of the major fronts of the ACC (the Sub-Antarctic Front or SAF, and the Polar Front or PF), velocity of deep (lower Circumpolar Deep Water) and intermediate (the Antarctic Intermediate Water or AAIW) water masses, among other major oceanographic events that occurred since the Last Glacial Maximum (LGM) up to the establishment of the current oceanographic regime around 10 Ka. The AAIW is particularly interesting in the present context, given that it flows at intermediate depths of approximately 700 to 1300 m [25], in a similar depth range as the collection sites of the samples analysed here (443–1712 m). Bostock et al. [24] defined the LGM for the Australian-New Zealand Southern Ocean region as having occurred from 21 to 18 Ka. During the most recent interglacial and LGM (30–18 Ka) the trajectory of the ACC (including STF, SAF, and PF) was located north of the current position [26], and the strength of the AAIW increased, flowing from north to south and vice versa [24]. Additionally, the Campbell Gyre intensified, which allowed a deep mixing of waters over the Campbell Plateau coupled with strong flow that reached the Chatham Rise [26, 27]. Both the Campbell Plateau and Chatham Rise host high abundances of *T. maia*. Additional evidence based on actual and LGM species distribution models suggests that the Tasman and Campbell Plateau, as well as other sub-Antarctic areas, were suitable as potential refuges during the LGM for the deep-sea shrimp *Nematocarcinus lanceopes*, restricting these Antarctic benthic organisms to higher latitudes [28]. Rapid changes in flow trajectories and intensities of the currents may have allowed higher connectivity among populations during the LGM, as evidenced by the presence of shared haplotypes between some distant populations.

While recent migration is indicated by shared haplotypes, we found high population divergence between northern and southern localities, clearly suggesting a barrier. On the other hand, the two least supported migration models were the ones that hypothesized an ACC barrier. Migration analyses would seem to suggest no barrier, but it is important to see barriers as a continuum [29]. This continuum is determined by physically and ecologically mediated disruptions in the range of a species. Within this continuum, ‘hard’ barriers are single physical features where dispersal is nil or very infrequent, and ‘soft’ barriers are general regions that experience climatic oscillations or exhibit ecological gradients where dispersal is not uncommon [29]. Soft barriers can also be seen as ‘filters’ acting only on selected species rather than impassable absolute barriers [30]. We thus conclude that the ACC is a ‘soft barrier’ that has suffered oscillations in position and velocity of some of its associated fronts and currents throughout its history. The ACC also exhibits environmental gradients in temperature and salinity in each front. Therefore, the ACC is capable of separating and structuring primnoid bottlebrush octocoral populations, while allowing bursts of limited gene flow.

Antarctica’s closest continental neighbor is South America, where the Antarctic Peninsula and Patagonia are separated by the Drake Passage, a channel that spans just 900 km with a depth of 4500 m [31]. This particular point has received considerable attention in biogeographical studies because of the short geographic distance between continental platforms, the common geological origin, and the presence of shared species (e.g., [1, 12, 32–34]). However, the ACC clearly acts as a barrier to gene flow for other species, such as the bottlebrush octocorals studied here, along with benthic organisms of several phyla, including brooding and spawning species, and deep- and shallow-water species [4, 10–13]. In some cases, samples from Antarctica and South America are highly diverged showing reciprocal monophyly, which suggests isolation, lack of gene flow between northern and southern populations as well as potentially cryptic speciation [10], as found here for brooder bottlebrush octocorals.

### Onset of the ACC versus time of diversification of the primnoid bottlebrush octocorals

The phylogenetic analyses show the population from Antarctica as the earliest divergent lineage, which is also the population with the highest genetic diversity. While source populations are often expected to have higher genetic diversity [35], using comparative methods we could not find support for any particular population being the ancestor. The origin of the ACC, dated around 25 Ma [8], likely did not drive the basal split of *T. maia*, which took place at some point during the Miocene (6.6–20.3 Ma), unless the process was particularly slow. A more likely driver of diversification in these bottlebrush corals was a shift in the trajectory of the ACC to north of New Zealand around the Middle Miocene [36]. This event likely allowed certain Antarctic organisms to disperse northward via the Macquarie Ridge, as hypothesized for notothenid fishes [36]. Taking into account the limited dispersal potential of brooding octocorals [37], the Macquarie Ridge may have also been the route connecting southern and northern populations of primnoid bottlebrush octocorals (Fig. 2b). During the Early Pliocene the ACC returned to its original position [36], and even though Antarctica and the Macquarie Ridge had already split, this new placement of the ACC between the respective populations could have helped sustaining the isolation of the northern populations. This shift southward may have allowed further diversification of the northern populations under different oceanographic regimes.

During the Middle Miocene, changes in the trajectories and intensities of oceanic currents were common and affected worldwide oceanographic patterns [38, 39]. As for the primnoid bottlebrush octocorals, these changes also had a great effect on the diversification of other corals and deep-sea dwellers. For example, the pattern of dispersal for the deep-sea octocoral *Paragorgia arborea* matches the Miocene ocean circulation model [35]. The deep-sea shrimp of the family Bresiliidae inhabit hydrothermal vents and hydrocarbon seeps, and their probable time of radiation has been placed in the Miocene around 20 Ma [40]. Moreover, during the Middle Miocene, between 13 and 16 Ma, there was a faunal exchange in foraminiferans involving extinction and speciation events, and changes in species’ distributional patterns [41]. All these changes during the Middle Miocene were probably associated with shifts in strength, chemical and physical properties of water masses, and the reorganizations of intermediate and deep ocean circulation that occurred as a result of the Antarctic glacial expansion [41–44]. Therefore it is not surprising that the diversification of the Southern Ocean and Southern Pacific primnoid bottlebrush octocorals took place at this time.

Time estimates, as the ones presented here, should be treated with caution whenever there are few strong calibration points [45]. We consider that using fossils from taxa that are closely related to Primnoidae would give a more reliable estimate of divergence time and, although increasing the number of calibration points would improve the precision of the divergence time estimates [46], no additional fossils with strong paleontological evidence were available. Two other studies have tried to estimate diversification times in primnoid octocorals [47, 48], but used older fossils that are more distantly related to Primnoidae or are unclear in their phylogenetic placement [49]. Using older fossils such as the ones employed in previous studies would have clearly pushed back the diversification time estimates in bottlebrush octocorals, an outcome that could also be expected if older fossils are found for Keratoisidinae or even for Primnoidae. The consequences that new fossils may bring on the diversification estimates presented here are uncertain, as with any other study that relies on calibration methods, hence the caution in using and interpreting the dates. However, we consider our estimates to be robust given our fossil selection and the priors used on this fossil (see Methods).

### Biodiversity implications

Genetic analyses clearly showed substantial population structure, often with monophyletic regional assemblages. These two results may support a case of cryptic speciation, but further taxonomical evaluation is needed. Due to recent migration events, some clades exhibit sympatry, as is the case of New Zealand and the Macquarie Ridge, where there is no apparent ecological split, at least in terms of depth. Additional information regarding the ecological preferences for each population would aid in further taxonomical classification, however such studies are methodologically challenging given the depth of the study sites. Currently, we rely on morphological characters from the whole colony, polyps, and sclerites to define and distinguish octocoral species [50]. Based on preliminary gross morphological analyses of the colonies, we have found differences in branching pattern and colour (Fig. 1). However at a microscopic level, we observe differential texture of the opercular scales, i.e., the sclerites that cover the tentacles of the polyps when retracted.

As previously mentioned, amongst the Antarctic population there are colonies that have the characters of *Tokoprymno maia* and others that resemble *Thouarella*. The general colony branching pattern of those resembling *Thouarella* is like that of *Tokoprymno*, while the polyp form and the shape and arrangement of the sclerites clearly suggest the samples belong to the species *Thouarella viridis* (apart from the colour of the colony and the absence of ridges on the coenenchymal sclerites). *Thouarella* is also a deep-sea brooding primnoid octocoral [37], which is commonly found in the Southern Ocean at depths around 400 m [51]. Some species of *Thouarella* share with the genus *Tokoprymno* the bottlebrush colony shape, polyps arranged singly, and the presence of a keel on the opercular scales [52]. Until recently, one of the major characters used to distinguish these two genera was the inclination or folding of the marginals over the bases of the operculars [37], but Taylor et al. [51] showed this is a species-specific character present in *Thouarella*, and many species have the marginals standing straight up or extensively flared, trumpet-like. Another character is that in *Thouarella* spp. the polyps are often angled distal resulting in a shorter adaxial side bearing a smaller number of sclerites, a character generally not encountered in *Tokoprymno*. Further, in *Thouarella* the polyps commonly have too small a circumference to accommodate the 8 marginals, resulting in them becoming arranged in two series of 4. But this is not consistent and some species have more or less only one ring of marginals, as is generally the case in *Tokoprymno*. Additionally, *Thouarella* is a polyphyletic group as shown in a recent paper by Taylor and Rogers [48], where some lineages are more closely related to *Tokoprymno*. This recent finding corroborated the systematic closeness of these two groups and the need for further taxonomic revision.

## Conclusions

This study represents the first assessment of phylogeographical patterns in deep-sea octocorals in the South Pacific and Southern Ocean. Our study system revealed that the ACC may be considered as a soft barrier, where it is possible to hypothesize recent migration. The ACC fluctuations in latitudinal positioning during the Miocene likely contributed to the diversification of these octocorals. Future analyses should include other types of genetic markers, such as microsatellites and SNPs, to more finely estimate gene flow, and evaluate possible admixture. Additional comparative phylogeographical data from different octocoral species, including both brooders and broadcast spawners, are needed to further corroborate the status of the ACC as a soft or filter barrier for these benthic deep-sea organisms.

## Methods

### Sample collection

We obtained dry and 96 % ethanol-preserved coral tissues from the National Institute of Water and Atmospheric Research (NIWA) Invertebrate Collection, the Museum of New Zealand Te Papa Tongarewa in Wellington, New Zealand, and from the Commonwealth Scientific and Industrial Research Organization (CSIRO) in Hobart, Tasmania. The samples were collected in a series of expeditions that date from 1961 to 2010, around New Zealand, Tasmania and the Ross Sea in Antarctica (Fig. 2 and Additional file 6). All samples were collected at depths between 443 and 1712 m through bottom trawling (benthic sled) and bottom longline. Most samples were bycatch and only a few came from research cruises that targeted whole benthic community diversity, thus the small sample size of octocorals. Additional sequences used here as outgroups were obtained from GenBank (Additional file 6), and see below.

### DNA extraction, amplification and sequencing

Using dry and ethanol preserved coral tissue, we extracted genomic DNA following a CTAB protocol [53]. After several attempts at DNA extraction, including the use of modified protocols, for some samples it was impossible to obtain high qualities and quantities of DNA. We successfully extracted DNA for 80 samples, which was resuspended in tris-ethylenediaminetetraacetic acid (TE) buffer and diluted to 20 ng/μl for use in PCR. Internal transcribed spacer 2 (ITS2) sequences and partial 28S rDNA sequences were obtained independently. For ITS2 we used primers 5.8S-436: 5′-AGCATGTCTGTCTGAGTGTTGG-3′ and 28S-663: 5′-GGGTAATCTTGCCTGATCTGAG-3′ [54], and for partial 28S we used primers F635sq: 5′-CCGTCTTGAAACACGGACC-3′ and R1411sq: 5′-GTTGTTACACACTCCTTAGCGG-3′ [55] that amplifies the D region (D2-D18) following the *Saccharomyces cerevisiae* LSU secondary structure model [56]. PCR profiles and primers are described in Aguilar and Sánchez [54] and Medina et al. [55], respectively. PCR products were purified using ExoSAP-IT (USB) and sent to Macrogen (Korea) where the products were Sanger-sequenced directly in both directions. Additional file 6 lists all samples, museum voucher numbers and corresponding GenBank accession numbers.

ITS2 and 28S belong to the nuclear ribosomal DNA (rDNA) marker family, which commonly consists of several tandem repeats [57]. These repeats do not evolve independently and are homogenized through a phenomenon called concerted evolution [58]. The rate of concerted evolution apparently varies among lineages of corals [59], and in some cases is high enough to completely homogenize the variation of rDNA repeats within individuals [57]. When rates of concerted evolution are slow, the number of copies within an individual could be as high as several hundred or even thousands [57, 60]. Genes belonging to the rDNA family have been widely used for corals and other marine benthic organisms in phylogenetic studies [61–64], in hybridization studies [65, 66], species delimitation purposes [61, 67, 68], and in population genetics studies [35, 69]. In terrestrial organisms, such as plants and insects, rDNA markers have also been used for population analyses [70, 71]. The use of rDNA genes in coalescent analyses has been discussed given the possibility of high copy numbers that would reduce the detection of population structure [72]. However, some organisms exhibit no intra-genomic variation, making direct sequencing possible [54, 63, 69, 73]. Assuming that no observed intra-genomic variation (as evidenced by direct sequencing) means that complete homogenization of gene copies has been achieved, coalescent analyses could be applied (with caution) to rDNA markers. This assumption has been employed several times for other anthozoans [63, 68, 73, 74]. In our case, we did not find any evidence whatsoever of multiple copies of rDNA genes within individuals of the deep-sea bottlebrush octocorals, as shown by the ease of direct sequencing and clean sequence chromatograms (i.e., no background peaks).

### Phylogenetic analyses

Complementary DNA chromatograms were assembled into consensus sequences using Geneious version R 8.1 (http://www.geneious.com, [75]). ITS2 and 28S DNA sequences were aligned independently using MUSCLE [76] and then concatenated for all further analyses, as these two genes are assumed to be in close physical linkage. Uncorrected p-distances were calculated in MEGA v6 [77] for each nuclear locus independently and the concatenated dataset.

Informative outgroup taxa were unavailable for both gene regions. GenBank contained data on some *Tokoprymno* that had ITS2 sequences but not 28S, and vice versa. Therefore we inferred an unrooted tree and used midpoint rooting for clearer interpretation. Topologies were inferred under the Maximum Likelihood (ML) and Bayesian Inference (BI) approaches. The optimal model of nucleotide substitution for the concatenated dataset was selected using PALM- Phylogenetic reconstruction by Automatic Likelihood Model selector [78], based on the Akaike Information Criterion (AIC). ML analyses were run under the TIMef + Γ model with 1000 bootstrap replicates in Garli v. 2.0 [79] implemented in the Cipres Science Gateway [80]. For BI, the evolutionary model was selected using jModeltest version 2.1.4 [81] via AIC evaluating only those models implemented in BEAST version 1.8.2 [82]. An unpartitioned phylogenetic analysis was then run under the HKY+ Γ model, consisting of 10 million Markov chain Monte Carlo generations and a burn-in of 25 %. Convergence and mixing were assessed using Tracer version 1.6 [83] by examining log-likelihood values across generations and ensuring that post-burn-in samples yielded an effective sample size (ESS) of >200 for all parameters.

### Phylogeographical analyses

The concatenated sequences were used for testing panmixia among sampling sites. Accordingly, we applied the Bayes Factor approach based on a comparison of marginal likelihood ratios [84], as implemented in MIGRATE-N version 3.6 [23]. These models were evaluated by taking into account the results obtained from the phylogenetic analyses (see previous section), which showed a subdivision between samples from New Zealand into two distinct populations: New Zealand and Macquarie Ridge. The models were run allowing for full migration between localities and included the following hypotheses: I) all localities as a panmictic population; II) a three-population model that included Tasmania, New Zealand (with Macquarie Ridge) and Antarctica; III) a four-population model that included Tasmania, New Zealand, Macquarie Ridge, and Antarctica (Additional File 1). MIGRATE-N was run with four Markov chains with a static heating scheme, 50,000 recorded steps, a burn-in of 5000 across 20 independent runs. Population sizes were not constrained, and we used uniform priors for the population mutation rate (θ) and the mutation-scaled migration rate (M). Given that there are no reports for nuclear mutation rates (μ) in octocorals, we used the default value. We report the log Bayes Factor (LBF) approximated by thermodynamic integration with Bézier approximation as suggested by Beerli and Palczewski [85]. As seen in the Results, the four-population model was the best-supported, therefore all subsequent analyses assumed four populations (i.e., Tasmania, New Zealand, Macquarie Ridge, and Antarctica).

To visualize the relationship between the genealogy of haplotypes and geography, haplotype networks were inferred using the median joining algorithm as implemented in PopArt [86]. Patterns of genetic variation within each population were explored using a combination of tools. DnaSP v5.1 [87] was used to obtain estimates of number of haplotype (h), haplotype diversity (Hd), nucleotide diversity per site (π) [88], and Watterson’s estimator of the per-site population mutation rate (*θ*_W_) [89]. Genetic variation was partitioned into within- and among-population components using an analysis of molecular variance (AMOVA; [90]). The AMOVA, implemented in Arlequin 3.5.1.3 [91], evaluated *a priori* hierarchical groupings: I) all collecting localities as one population (i.e., no ACC barrier), and II) sub-Antarctic localities as one population compared to Antarctica (i.e., ACC as a barrier). Pairwise *F*_ST_ values [92] were calculated using DnaSP v. 5.1 to test the null hypothesis of panmixia between pairs of populations [93] with 10,000 permutations and a significance level of 0.05.

To evaluate possible asymmetries in migration rates between population pairs, we compared eight different migration models that enforce particular patterns of spatial population genetic structure for Tasmania, New Zealand, Macquarie Ridge, and Antarctica: I) full migration model between the four localities; II) migration from West to East; III) migration from East to West; IV) migration from North to South; V) migration from South to North; VI) a stepping-stone model; VII) ACC as a complete barrier between northern and southern populations, but allowing full migration among northern populations; VIII) ACC as a barrier and stepping-stone between northern populations (Table 4). The models were run in MIGRATE-N using a Bayes Factor approach through a thermodynamic integration with the same parameters as above.

### Estimating divergence times

To test whether the divergence time of southern and northern ACC populations predated the onset of the ACC, we estimated divergence times in BEAST version 1.8.2 [82]. There are no known fossils for *Tokoprymno* or *Thouarella*. Given that Isididae has been shown to be the sister clade to Primnoidae [94], for calibration purposes, we used as outgroup species (Additional file 6) seven bamboo corals from the Subfamily Keratoisidinae (*Lepidisis solitaria* [GenBank:FJ790910], *Acanella* sp. [GenBank:FJ790922], *Keratoisis hikurangiensis* [GenBank:FJ790941], *Keratoisis projecta* [GenBank:FJ790942], *Acanella weberi* [GenBank:FJ790943], *Isidella tentaculum* [GenBank:FJ790945], *Keratoisis magnifica* [GenBank:KT260062]), and an additional primnoid, *Calyptrophora japonica* [GenBank: EF090735]. As a temporal calibration constraint, we employed the oldest known fossil for the Keratoisidinae clade, i.e., *Keratoisis* sp. from the Otaian-Miocene, dated between 21.7 and 19.0 Ma (= *Keratoisis tangentis* Grant 1976 in [95]). The minimum age of this fossil was treated as a minimum constraint on the age of the stem group node using a log-normal distributed prior [96]. The lognormal distribution has been considered the most appropriate for summarizing paleontological information [97]. This statistical distribution (as well as the exponential distribution) assigns a non-zero probability to node ages approaching infinity [98]. The lognormal thus accommodates much older ages albeit with diminishing prior probabilities [99, 100]. Following Ho and Phillips [97], we selected the value of the standard deviation so that 95 % of the probability density lies between the minimum constraint and the oldest date of the geological range where the fossil was found (21.7 Ma). This same method has been used in the diversification estimates of other deep-sea octocoral groups such as Coralliidae [101] and *Callogorgia* [47].

To estimate the divergence times we used only the ITS2 region, which had available outgroup sequences. Bayesian MCMC analyses assumed a Birth-Death speciation tree prior. Having previously rejected a strict clock model (results not shown), divergence times were estimated using a relaxed molecular clock with log-normal uncorrelated rates. The analysis was run under a HKY+ Γ model twice to avoid searching only on local optima [102] and utilized 10^7^ generations and default heating values on three Metropolis-coupled chains. Trees and parameters were sampled every 1000 generations and the first 25 % of the samples were discarded as burn-in. To check for adequate convergence and confirm a satisfactory effective sample size (ESS > 200), Tracer v. 1.8., LogCombiner v. 1.8, and TreeAnnotator v. 1.8 were used to combine and summarize trees files, obtain a maximum clade credibility consensus tree, and calculate 95 % credibility intervals [82]. Finally we ran the analysis on an empty dataset, sampling from the prior distribution to evaluate the influence of the priors on the posterior distribution estimates [103, 104].

### Ancestral character state reconstruction

To establish the geographic origin of this group, we reconstructed the ancestral locality taking each of the four populations (see above) as a discrete character state. The analysis used the ITS2 topology obtained from the divergence estimation (see *Estimating divergence times*). However, for the ease of interpretation, we removed the outgroup clade using the drop.tip function from the package ‘ape’ for R [105]. The analysis was based on the maximum likelihood ancestral state reconstruction method and the stochastic character mapping using the packages ‘phytools’ [106] and ‘geiger’ [107] in R. For this purpose we employed a symmetric rate model, an empirical Q matrix, and 1000 simulations.

### Availability of supporting data

Sample information as museum codes and locations are provided as supporting information in the Additional file 6. DNA sequences for both gene regions can be found in GenBank and also listed in Additional file 6. 28S [GenBank: KT259907 - KT259986]; ITS2 [GenBank: KT259987 - KT260067].

## Additional files

Additional file 1:
**MIGRATE-N runs showing the log-probability of the data given the model (marginal likelihood).** The runs evaluated the following migration models: I) all localities form a single panmictic population; II) three-population model; III) four-population model. The first row represents the number of parameters used for a particular model. The second row shows the symbol specification for the migration matrix given to MIGRATE-N, where and asterisk represents an estimated parameter and a zero a non-estimated parameter. The third row provides the log marginal likelihood for each model obtained by thermodynamic integration. The forth row reports the log Bayes Factor (LBF). The row ‘Choice’ orders the models based on the LBF, and finally, the sixth row shows the probability for each model. Populations are labelled as follows: T for Tasmania, MQ for Macquarie Ridge, NZ for New Zealand, and A for Antarctica. (DOCX 126 kb) Additional file 2:
**MIGRATE-N output for the full model of migration between populations of the bottlebrush deep-sea octocorals.** This analysis was based on 80 samples and the two nuclear genes ITS2 and 28S for samples from the South Pacific and Southern Ocean. Values reported correspond to median values for the population mutation rate (θ) above the diagonal, with the mutation-scaled immigration rate (M) for each direction of migration below the diagonal with source populations as rows and receiving population as columns. Values in parenthesis show the 2.5–97.5 % credible interval for each reported value of θ and M. (DOCX 63 kb) Additional file 3:
**Ancestral character state reconstruction for the locality of the bottlebrush octocorals through a maximum likelihood approach.** This analysis was run under a symmetric rate model using the packages ‘phytools’ [107] and ‘geiger’ [108] in R. Pie diagrams at nodes represent probabilities for each state, and the colours correspond to the localities: red for Tasmania, green for New Zealand, orange for Macquarie Ridge, and blue for Antarctica. Notice that for nodes A, B, and C the ancestral locality reconstruction is unresolved. (DOCX 153 kb) Additional file 4:
**Ancestral character state reconstruction for the locality of the bottlebrush octocorals through a stochastic mapping approach.** This analysis was run under a symmetric rate model, an empirical Q matrix, and 1000 simulations using the packages ‘phytools’ [107] and ‘geiger’ [108] in R. The figure shows an example of nine reconstructions from a simulation of 1000 trees, where is evident the lack of resolution on the ancestral locality for node A, B, and C. The colours correspond to the localities: red for Tasmania, green for New Zealand, orange for Macquarie Ridge, and blue for Antarctica. (DOCX 319 kb) Additional file 5:
**Posterior probabilities of each state at three nodes, for the ancestral character state reconstruction.** Localities were reconstructed through an stochastic mapping approach. Node numbering corresponds to the same as Additional files 3 and 4. (DOCX 40 kb) Additional file 6:
**Samples used in this study.** Information includes the museum code, collection locality, and GenBank accession numbers for the two nuclear gene regions. Sequences that were not obtained in this study are indicated by and asterisk (*). (DOCX 118 kb)

## Abbreviations

28S: 28S ribosomal RNA
AAIW: Antarctic Intermediate Water
ACC: Antarctic Circumpolar Current
AIC: Akaike Information Criterion
AMOVA: Analysis of Molecular Variance
BI: Bayesian Inference
CI: Credible Interval
CSIRO: Commonwealth Scientific and Industrial Research Organization
CTAB: Cetyltrimethyl ammonium bromide
ESS: Effective Sample Size
*F*_*ST*_: F-statistic
h: number of haplotype
Hd: haplotype diversity
ITS2: internal transcribed spacer 2
Ka: Thousand years ago
LBF: log Bayes Factor
LGM: Last Glacial Maximum
m: immigration rate per generation
M: Mutation-scaled migration rates (M = m/μ)
Ma: Million years ago
ML: Maximum Likelihood
NIWA: National Institute of Water and Atmospheric Research
PCR: Polymerase chain reaction
PF: Polar Front
SAF: Sub-Antarctic Front
SST: sea surface temperature
TE: Tris-ethylenediaminetetraacetic acid buffer
π: nucleotide diversity per site
*θ*w: Watterson’s estimator of the per-site population mutation rate
μ: mutation rate
θ: Population mutation rate
