# Supplementary material for: The Antarctic Circumpolar Current as a diversification trigger for deep-sea octocorals
Source: BMC Evol Biol. 2016 Jan 4;16:2. doi: 10.1186/s12862-015-0574-z (PMC4700699; doi:10.1186/s12862-015-0574-z)
Supplement: Additional file 1: — MIGRATE-N runs showing the log-probability of the data given the model (marginal likelihood). The runs evaluated the following migration models: I) all localities form a single panmictic population; II) three-population model; III) four-population model. The first row represents the number of parameters used for a particular model. The second row shows the symbol specification for the migration matrix given to MIGRATE-N, where and asterisk represents an estimated parameter and a zero a non-estimated parameter. The third row provides the log marginal likelihood for each model obtained by thermodynamic integration. The forth row reports the log Bayes Factor (LBF). The row ‘Choice’ orders the models based on the LBF, and finally, the sixth row shows the probability for each model. Populations are labelled as follows: T for Tasmania, MQ for Macquarie Ridge, NZ for New Zealand, and A for Antarctica. (DOCX 126 kb) [file 12862_2015_574_MOESM1_ESM.docx]

**Additional File 1** **MIGRATE-N runs showing the log-probability of the data given the model (marginal likelihood).** The runs evaluated the following migration models: I) all localities form a single panmictic population; II) three-population model; III) four-population model. The first row represents the number of parameters used for a particular model. The second row shows the symbol specification for the migration matrix given to MIGRATE-N, where and asterisk represents an estimated parameter and a zero a non-estimated parameter. The third row provides the log marginal likelihood for each model obtained by thermodynamic integration. The forth row reports the log Bayes Factor (LBF). The row 'Choice' orders the models based on the LBF, and finally, the sixth row shows the probability for each model. Populations are labelled as follows: T for Tasmania, MQ for Macquarie Ridge, NZ for New Zealand, and A for Antarctica.

|  | **Model I** | **Model II** | **Model III** |
| --- | --- | --- | --- |
|  | 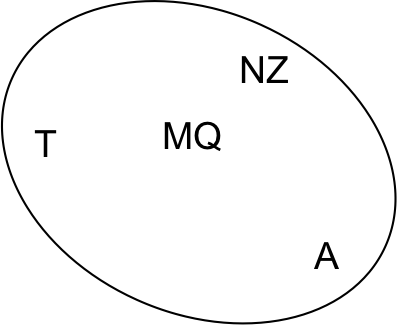 | 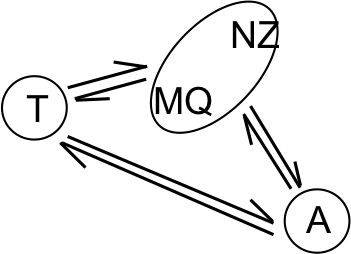 | 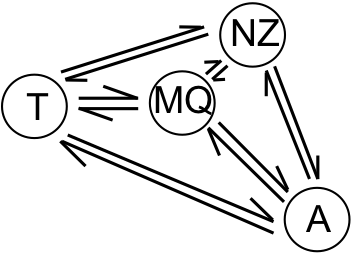 |
| **No. Parameters** | 1 | 9 | 16 |
| **Specification** | * | ********* | **************** |
| **Bezier lmL** | -5358.02 | -5276.73 | -5235.54 |
| **LBF** | -122.46 | -41.17 | 0.00 |
| **Choice** | 3 | 2 | 1 |
| **Posterior Prob.** | 6.55 E -54 | 1.319 E -18 | 1.00 |
|  |  |  |  |
