# Supplementary material for: The Antarctic Circumpolar Current as a diversification trigger for deep-sea octocorals
Source: BMC Evol Biol. 2016 Jan 4;16:2. doi: 10.1186/s12862-015-0574-z (PMC4700699; doi:10.1186/s12862-015-0574-z)
Supplement: Additional file 2: — MIGRATE-N output for the full model of migration between populations of the bottlebrush deep-sea octocorals. This analysis was based on 80 samples and the two nuclear genes ITS2 and 28S for samples from the South Pacific and Southern Ocean. Values reported correspond to median values for the population mutation rate (θ) above the diagonal, with the mutation-scaled immigration rate (M) for each direction of migration below the diagonal with source populations as rows and receiving population as columns. Values in parenthesis show the 2.5–97.5 % credible interval for each reported value of θ and M. (DOCX 63 kb) [file 12862_2015_574_MOESM2_ESM.docx]

**Additional File 2** **MIGRATE-N output for the full model of migration between populations of the bottlebrush deep-sea octocorals.** This analysis was based on 81 samples and the two nuclear genes ITS2 and 28S for samples from the South Pacific and Southern Ocean. Values reported correspond to median values for the population mutation rate (θ) above the diagonal, with the mutation-scaled immigration rate (M) for each direction of migration below the diagonal with source populations as rows and receiving population as columns. Values in parenthesis show the 2.5%-97.5% credible interval for each reported value of θ and M.

|  | Macquarie Ridge | Tasmania | New Zealand | Antarctica |
| --- | --- | --- | --- | --- |
|  | θ = 0.0890 (0.0641-0.1000) | θ = 0.0039 (0.0000-0.0162) | θ = 0.0234 (0.0032-0.0367) | θ = 0.0717 (0.0233-0.1000) |
| Macquarie Ridge | - | 24.3 (0.0-264.7) | 16.3 (0.0-92.0) | 27.7 (0.0-153.3) |
| Tasmania | 77.0 (0.0-364.0) | - | 281.0 (0.0-623.3) | 186.3 (0.0-441.3) |
| New Zealand | 48.3 (0.0-184.0) | 71.7 (0.0-216.7) | - | 64.3 (0.0-240.7) |
| Antarctica | 87.0 (0.0-265.3) | 98.3 (0.0-701.3) | 189.7 (0.0-600.7) | - |
