# Supplementary material for: The Antarctic Circumpolar Current as a diversification trigger for deep-sea octocorals
Source: BMC Evol Biol. 2016 Jan 4;16:2. doi: 10.1186/s12862-015-0574-z (PMC4700699; doi:10.1186/s12862-015-0574-z)
Supplement: Additional file 3: — Ancestral character state reconstruction for the locality of the bottlebrush octocorals through a maximum likelihood approach. This analysis was run under a symmetric rate model using the packages ‘phytools’ [107] and ‘geiger’ [108] in R. Pie diagrams at nodes represent probabilities for each state, and the colours correspond to the localities: red for Tasmania, green for New Zealand, orange for Macquarie Ridge, and blue for Antarctica. Notice that for nodes A, B, and C the ancestral locality reconstruction is unresolved. (DOCX 153 kb) [file 12862_2015_574_MOESM3_ESM.docx]

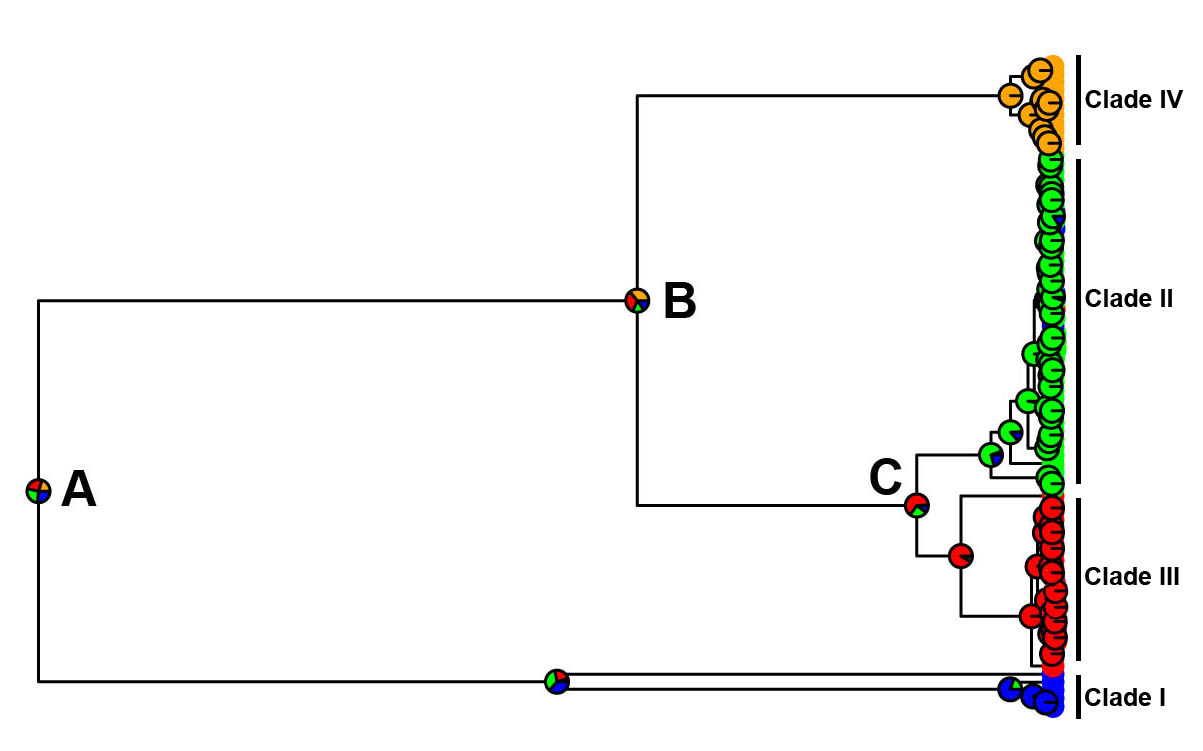


**Additional File 3** **Ancestral character state reconstruction for the locality of the bottlebrush octocorals through a maximum likelihood approach.** This analysis was run under a symmetric rate model using the packages 'phytools' [108] and 'geiger' [109] in R. Pie diagrams at nodes represent probabilities for each state, and the colours correspond to the localities: red for Tasmania, green for New Zealand, orange for Macquarie Ridge, and blue for Antarctica. Notice that for nodes A, B, and C the ancestral locality reconstruction is unresolved.

108. Revell LJ. Phytools: An R package for phylogenetic comparative biology (and other things). Methods in Ecology and Evolution. 2012;3:217-23.

109. Harmon LJ, Weir JT, Brock CD, Glor RE, Challenger W. GEIGER: investigating evolutionary radiations. Bioinformatics. 2008;24:129-31.
