# Supplementary material for: The Antarctic Circumpolar Current as a diversification trigger for deep-sea octocorals
Source: BMC Evol Biol. 2016 Jan 4;16:2. doi: 10.1186/s12862-015-0574-z (PMC4700699; doi:10.1186/s12862-015-0574-z)
Supplement: Additional file 5: — Posterior probabilities of each state at three nodes, for the ancestral character state reconstruction. Localities were reconstructed through an stochastic mapping approach. Node numbering corresponds to the same as Additional files 3 and 4. (DOCX 40 kb) [file 12862_2015_574_MOESM5_ESM.docx]

**Additional File 5** Posterior probabilities of each state at three nodes, for the ancestral character state reconstruction. Localities were reconstructed through an stochastic mapping approach. Node numbering corresponds to the same as Additional File 5 and 6.

| Node | Macquarie Ridge | Tasmania | New Zealand | Antarctica |
| --- | --- | --- | --- | --- |
| A | 0.211 | 0.269 | 0.235 | 0.285 |
| B | 0.347 | 0.320 | 0.173 | 0.160 |
| C | 0.009 | 0.618 | 0.264 | 0.109 |
