# Supplementary material for: The Antarctic Circumpolar Current as a diversification trigger for deep-sea octocorals
Source: BMC Evol Biol. 2016 Jan 4;16:2. doi: 10.1186/s12862-015-0574-z (PMC4700699; doi:10.1186/s12862-015-0574-z)
Supplement: Additional file 6: — Samples used in this study. Information includes the museum code, collection locality, and GenBank accession numbers for the two nuclear gene regions. Sequences that were not obtained in this study are indicated by and asterisk (*). (DOCX 118 kb) [file 12862_2015_574_MOESM6_ESM.docx]

**Additional File 6** **Samples used in this study**. Information includes the museum code, collection locality, and GenBank accession numbers for the two nuclear gene regions. Sequences that were not obtained in this study are indicated by and asterisk (*).

|  |  | |  | | | **GenBank Acc. Number** | |
| --- | --- | --- | --- | --- | --- | --- | --- |
| **Museum** | **Museum Code** | | **Locality** | | | **ITS2** | **28S** |
| CSIRO | 0207 12-19 | | Tasmania | | | KT260056 | KT259917 |
| CSIRO | 0207 22-35 | | Tasmania | | | KT260057 | KT259916 |
| CSIRO | 0207 26-6 | | Tasmania | | | KT260053 | KT259920 |
| CSIRO | 0207 34-9 | | Tasmania | | | KT260054 | KT259919 |
| CSIRO | 0207 37-6 | | Tasmania | | | KT260055 | KT259918 |
| CSIRO | 22-32 | | Tasmania | | | KT259987 | KT259986 |
| CSIRO | 9717 K 13-28 | | Tasmania | | | KT259988 | KT259985 |
| CSIRO | J2 382 15-1 | | Tasmania | | | KT260059 | KT259914 |
| CSIRO | J2 386 15-2 | | Tasmania | | | KT260060 | KT259913 |
| CSIRO | J2 389 6-5 | | Tasmania | | | KT260057 | KT259915 |
| CSIRO | J2 389 8-10 | | Tasmania | | | KT260061 | KT259912 |
| NIWA | NIWA 9722 | | New Zealand | | | KT259989 | KT259984 |
| NIWA | NIWA 11320 | | New Zealand | | | KT259990 | KT259983 |
| NIWA | NIWA 11321 | | New Zealand | | | KT259991 | KT259982 |
| NIWA | NIWA 11326 | | New Zealand | | | KT259992 | KT259981 |
| NIWA | NIWA 11328 | | New Zealand | | | KT259993 | KT259980 |
| NIWA | NIWA 11331 | | New Zealand | | | KT259994 | KT259979 |
| NIWA | NIWA 11332 | | Tasmania | | | KT259995 | KT259978 |
| NIWA | NIWA 11335 | | Tasmania | | | KT259996 | KT259977 |
| NIWA | NIWA 11336 | | Tasmania | | | KT259997 | KT259976 |
| NIWA | NIWA 11339 | | Tasmania | | | KT259998 | KT259975 |
| NIWA | NIWA 11341 | | Tasmania | | | KT259999 | KT259974 |
| NIWA | NIWA 11344 | | New Zealand | | | KT260000 | KT259973 |
| NIWA | NIWA 14371 | | Tasmania | | | KT260001 | KT259972 |
| NIWA | NIWA 14642 | | New Zealand | | | KT260002 | KT259971 |
| NIWA | NIWA 14682 | | New Zealand | | | KT260003 | KT259970 |
| NIWA | NIWA 14684 | | New Zealand | | | KT260004 | KT259969 |
| NIWA | NIWA 15494 | | Tasmania | | | KT260005 | KT259968 |
| NIWA | NIWA 26886 | | New Zealand | | | KT260006 | KT259967 |
| NIWA | NIWA 26890 | | New Zealand | | | KT260007 | KT259966 |
| NIWA | NIWA 26891 | | New Zealand | | | KT260008 | KT259965 |
| NIWA | NIWA 39606 | | New Zealand | | | KT260009 | KT259964 |
| NIWA | NIWA 39717 | | New Zealand | | | KT260010 | KT259963 |
| NIWA | NIWA 39753 | | New Zealand | | | KT260011 | KT259962 |
| NIWA | NIWA 39786 | | Macquarie Ridge | | | KT260012 | KT259961 |
| NIWA | NIWA 39831 | | New Zealand | | | KT260013 | KT259960 |
| NIWA | NIWA 40018 | | New Zealand | | | KT260014 | KT259959 |
| NIWA | NIWA 40035 | | New Zealand | | | KT260015 | KT259958 |
| NIWA | NIWA 40457 | | Macquarie Ridge | | | KT260016 | KT259957 |
| NIWA | NIWA 40458 | | New Zealand | | | KT260017 | KT259956 |
| NIWA | NIWA 40480 | | Macquarie Ridge | | | KT260018 | KT259955 |
| NIWA | NIWA 40542 | | Macquarie Ridge | | | KT260019 | KT259954 |
| NIWA | NIWA 40545 | | New Zealand | | | KT260020 | KT259953 |
| NIWA | NIWA 40667 | | Macquarie Ridge | | | KT260021 | KT259952 |
| NIWA | NIWA 40727 | | Macquarie Ridge | | | KT260022 | KT259951 |
| NIWA | NIWA 40730 | | Macquarie Ridge | | | KT260023 | KT259950 |
| NIWA | NIWA 40850 | | New Zealand | | | KT260024 | KT259949 |
| NIWA | NIWA 40978 | | Macquarie Ridge | | | KT260025 | KT259948 |
| NIWA | NIWA 41111 | | Macquarie Ridge | | | KT260027 | KT259946 |
| NIWA | NIWA 41105 | | Macquarie Ridge | | | KT260026 | KT259947 |
| NIWA | NIWA 42521 | | New Zealand | | | KT260028 | KT259945 |
| NIWA | NIWA 42569 | | New Zealand | | | KT260029 | KT259944 |
| NIWA | NIWA 42570 | | Macquarie Ridge | | | KT260030 | KT259943 |
| NIWA | NIWA 53275 | | New Zealand | | | KT260031 | KT259942 |
| NIWA | NIWA 53489 | | New Zealand | | | KT260032 | KT259941 |
| NIWA | NIWA 61408 | | New Zealand | | | KT260033 | KT259940 |
| NIWA | NIWA 61409 | | New Zealand | | | KT260034 | KT259939 |
| NIWA | NIWA 64812 | | New Zealand | | | KT260035 | KT259938 |
| NIWA | NIWA 66098 | | New Zealand | | | KT260036 | KT259937 |
| NIWA | NIWA 66101 | | New Zealand | | | KT260037 | KT259936 |
| NIWA | NIWA 66109 | | New Zealand | | | KT260038 | KT259935 |
| NIWA | NIWA 66142 | | New Zealand | | | KT260039 | KT259934 |
| NIWA | NIWA 66160 | | New Zealand | | | KT260040 | KT259933 |
| NIWA | NIWA 66178 | | New Zealand | | | KT260041 | KT259932 |
| NIWA | NIWA 66309 | | New Zealand | | | KT260042 | KT259931 |
| NIWA | NIWA 68213 | | Antarctica | | | KT260043 | KT259930 |
| NIWA | NIWA 68214 | | New Zealand | | | KT260044 | KT259929 |
| NIWA | NIWA 68215 | | Antarctica | | | KT260045 | KT259928 |
| NIWA | NIWA 68216 | | Antarctica | | | KT260046 | KT259927 |
| NIWA | NIWA 68217 | | Antarctica | | | KT260047 | KT259926 |
| NIWA | NIWA 68218 | | Antarctica | | | KT260048 | KT259925 |
| NIWA | NIWA 68219 | | Antarctica | | | KT260049 | KT259924 |
| NIWA | NIWA 68220 | | Antarctica | | | KT260050 | KT259923 |
| NIWA | NIWA 68221 | | Antarctica | | | KT260051 | KT259922 |
| NIWA | NIWA 68222 | | Antarctica | | | KT260052 | KT259921 |
| CSIRO | SS01 97-41 | | Tasmania | | | KT260063 | KT259911 |
| CSIRO | SS0207 12-19 | | Tasmania | | | KT260065 | KT259909 |
| CSIRO | SS0207 13-33 | | Tasmania | | | KT260066 | KT259908 |
| CSIRO | SS0207 25-13 | | Tasmania | | | KT260067 | KT259907 |
| CSIRO | SS0207 26-6 | | Tasmania | | | KT260064 | KT259910 |
|  |  | |  | | |  |  |
| Outgroups | |  | | Species |  |  |  |
|  |  | | *Calyptrophora japonica ** | | | EF090735 | N/A |
|  |  | | *Lepidisis solitaria** | | | FJ790910 | N/A |
|  |  | | *Acanella* sp. * | | | FJ790922 | N/A |
|  |  | | *Keratoisis hikurangiensis ** | | | FJ790941 | N/A |
|  |  | | *Keratoisis projecta ** | | | FJ790942 | N/A |
|  |  | | *Acanella weberi ** | | | FJ790943 | N/A |
|  |  | | *Isidella tentaculum ** | | | FJ790945 | N/A |
| NIWA | NIWA14375 | | *Keratoisis magnifica* | | | KT260062 | N/A |
|  |  | |  | | |  |  |
